# Do soldiers seek more mental health care after deployment? Analysis of mental health consultations in the Netherlands Armed Forces following deployment to Afghanistan

**DOI:** 10.3402/ejpt.v5.23667

**Published:** 2014-08-14

**Authors:** Elisabeth (Liesbeth) M. Taal, Eric Vermetten, Digna (Anneke) J. F. van Schaik, Tjalling Leenstra

**Affiliations:** 1 Military Health Care Expertise and Coordination Center, Netherlands Ministry of Defense, Doorn, The Netherlands; 2 Military Mental Health—Research Center, Netherlands Ministry of Defense, Utrecht, The Netherlands; 3 Department Psychiatry, Leiden University Medical Center, Leiden, The Netherlands; 4 Department of Psychiatry, VU University Medical Centre Amsterdam, Amsterdam, The Netherlands; 5 EMGO Institute for Health and Care Research (EMGO +), VU University Medical Centre Amsterdam, Amsterdam, The Netherlands

**Keywords:** Military personnel, deployment, mental disorders, mental health care, service utilization, combat related stress disorders, hazard ratios

## Abstract

**Background:**

Military deployment to combat zones puts military personnel to a number of physical and mental challenges that may adversely affect mental health. Until now, few studies have been performed in Europe on mental health utilization after military deployment.

**Objective:**

We compared the incidence of mental health consultations with the Military Mental Health Service (MMHS) of military deployed to Afghanistan to that of non-deployed military personnel.

**Method:**

We assessed utilization of the MMHS by the full cohort of the Netherlands Armed Forces enlisted between 2008 and 2010 through linkage of mental health and human resource information systems.

**Results:**

The total population consisted of 50,508 military (18,233 deployed, 32,275 non-deployed), who accounted for 1,906 new consultations with the MMHS. The follow-up was limited to the first 2 years following deployment. We observed higher mental health care utilization in deployed vs. non-deployed military personnel; hazard ratio (HR), adjusted for sex, military branch and time in service, 1.84 [95% CI 1.61–2.11] in the first and 1.28 [1.09–1.49] in the second year after deployment. An increased risk of adjustment disorders (HR 2.59 [2.02–3.32] and 1.74 [1.30–2.32]) and of anxiety disorders (2.22 [1.52–3.25] and 2.28 [1.50–3.45]) including posttraumatic stress disorder (5.15 [2.55–10.40] and 5.28 [2.42–11.50]), but not of mood disorders (1.33 [0.90–1.97] and 1.11 [0.68–1.82]), was observed in deployed personnel in the first- and second-year post-deployment, respectively. Military personnel deployed in a unit with a higher risk of confrontation with potentially traumatic events had a higher HR (2.13 [1.84–2.47] and 1.40 [1.18–1.67]).

**Conclusions:**

Though absolute risk was low, in the first and second year following deployment to Afghanistan there was an 80 and 30% higher risk for mental health problems resulting in a consultation with the Dutch MMHS compared to military never deployed to Afghanistan. These observations underscore the need for an adequate mental health infrastructure for those returning from deployment.

In recent years, the Netherlands Armed Forces have taken part in the International Security Assistance Force (ISAF) mission in Afghanistan. Since 2002, more than 25,000 Dutch soldiers have been deployed to Afghanistan (Netherlands Institute of Military History, 2012). Participation in a military deployment is frequently associated with a higher risk of developing mental health problems. Frequencies of operational stress injuries, combat stress reactions, symptoms of posttraumatic stress, depression, fatigue, and alcohol abuse in military personnel following deployment have been reported (Brown, Williams, Bray, & Hourani, 2012; Fear et al., 2010; Hoge, Auchterlonie, & Milliken, 2006; Hoge et al., 2004; Milliken, Auchterlonie, & Hoge, 2007; Rona et al., 2012; Vasterling et al., 2010; Wells et al., 2010). Research studies demonstrated a two- to four-fold increase in life-time prevalence of posttraumatic stress disorder (PTSD) among US combat veterans as compared to the general US population (Richardson, Frueh, & Acierno, 2010). Recent studies in troops deployed to Iraq and Afghanistan also confirmed that combat exposure as well as exposure to deployment-related stressors, including stressors like being away from family and friends, resulted in stress sensitization (Smid et al., 2013), contributing to an increased risk for a variety of mental health problems (Hoge et al., 2004, 2006; Milliken et al., 2007; Rona et al., 2009).

As can be seen in various studies in this volume, the prevalence vary considerably, depending on the study method used or study population assessed. Some studies only found increases in mental health problems in specific groups like Reserve Forces (Iversen et al., 2009). Following deployment to recent military operations in Iraq and Afghanistan, prevalence rates in US and UK military personnel for the development of symptoms of PTSD that have been reported were 4–13%; for major depressive disorders 3.7–7.9%; for anxiety disorders 4.5–7.9%, and for alcohol abuse 13–26% (Fear et al., 2010; Hoge et al., 2004; Hotopf et al., 2006; Iversen et al., 2009).

In Dutch military personnel deployed to Iraq, the prevalence of symptom of PTSD was found to be less than 5%. It was interesting to find that the mean level of symptoms of PTSD in this group remained relatively stable before and after deployment (Engelhard et al., 2007). However, in recent analysis of a large cohort of Dutch military personnel prior to deployment and monitored up to 2 years after returning from Afghanistan, the prevalence of symptoms of PTSD, depression, anxiety, somatic complaints, sleeping problems, and fatigue was found to increase after deployment when compared with the rates found prior to deployment (Smid et al., 2013; Reijnen et al., 2014).

Not only for the Dutch military health care organization but also from a political perspective, it is important to quantify the magnitude of the relationship between the development of mental health utilization in military personnel and deployment, as this could have important policy consequences (Tweede Kamer der Staten-Generaal, 2010, 2011).

Dutch military personnel are required to consult a primary care physician in the military health care system in case of disease or general health problems. They can request and receive help outside of this system, but this will not be reimbursed, and it has to be consumed in free time. In case of mental problems, the primary care physician or an occupational social worker can refer the individual to the four regional branches of the Military Mental Health Services (MMHS) in the Netherlands Armed Forces. Typically, the service allows rapid consultation within the first week after referral. The Forces have a structure that is comparable to other NATO partners, with Army, Navy and Air Force, and MMHS provides care to all branches. Since 2008, the MMHS has used an electronic health information system (HIS), which contains a DSM IV diagnosis for all patients. In this study, we used this database to investigate the number of new consultations and diagnoses with the MMHS for different groups of military personnel.

The goal of this study was to assess the incidence of mental health problems resulting in a consultation with the MMHS in military personnel that were recently deployed to Afghanistan in comparison to military personnel that were not deployed within the previous 3 years. This was studied both by assessment of consultations with the MMHS, as well as by assessment of specific DSM IV diagnoses.

## Method

### Study population

Military personnel in active duty between September 1, 2008, and November 15, 2010, or part of this period were included in the study. Reserve forces were excluded, because this group is not required to visit a military health care provider and will usually see civil health professionals. Military personnel already in treatment with the MMHS preceding the observation period (from January till August 2008, or part of this period) were excluded. Military personnel recently (after 2005) deployed to countries other than Afghanistan (e.g., Bosnia, Chad) were also excluded. In addition, military personnel deployed to Afghanistan for frequent short visits of <30 days were excluded.

### Study data

Routinely collected data were extracted from a HIS and a human resource management system. No additional information was obtained by structured interviews or self-assessments so for the accuracy of the data we relied on the careful performed assessment in by the clinicians using the database. For the collection of the data we did not interfere with the clinical diagnostic process.

### Health information system

From 2008 the MMHS used a HIS that contained an electronic appointment system. Appointments were labeled with codes that provided information about diagnosis and treatment. These codes referred to reimbursement for the individual and contained information about diagnosis, categorized according to the DSM IV classification system. For example, the main diagnosis (e.g., Major depressive disorder, single episode) and the group of diagnoses (e.g., Mood disorders) were available. All mental health professionals of the MMHS have been trained in using the DSM IV coding system. The MMHS has been implementing this information system since January 2008 and as from September 2008 reliable data were available.

### Human resource management system

The human resource management system that was implemented by the Netherlands Ministry of Defense in 2004 supports a number of human resources and business operations. Details about employees, contract type, and military rank and function were routinely registered. For this study, data were available from 2006 onward. Data relevant to this study were date of birth, sex, rank, hire date, date of leaving the military, and details about the participation in a military mission (location, period, military unit, function).

### Ethical issues

To guarantee the privacy of the military personnel, personal information were anonymized and encoded when databases were merged. To prevent indirect traceability of data to individuals, personal characteristics were recoded to aggregate categories. For example, data on specific functions or rank were replaced by more general categories like soldier, non-commissioned- or staff-officer. The data manager of MMHS merged the personnel file with diagnostic data by means of the Citizen Service Number (in Dutch: Burger Service Nummer, BSN). A new unique identification number was assigned to every individual and the BSN was removed. The coding key was not saved.

This study did not require investigational review board approval, as it used available routinely collected data. The Surgeon General of the Dutch Ministry of Defense and the Commanding Officer of the Dutch Military Mental Health Organization commissioned this study. Because this study was anonymized, routinely obtained medical data were used and individual consent was not required. Patients of the MMHS are routinely informed that anonymized patient data may be used for research aimed to improve health.

### Data analysis

The primary exposure variable was deployment to Afghanistan. Deployment was defined as having served a minimum of 30 consecutive days in a military deployment. The typical duration of deployment varies between 4 and 6 months. Once a veteran had been deployed to Afghanistan, he was regarded as being “exposed” for the duration of follow-up. A consultation with the MMHS could only have occurred after return from deployment. In the case a military person was repatriated due to mental disorders, the first consultation with the MMHS would have been registered following return to the Netherlands.

The primary outcome was a registered consultation with the MMHS. A consultation was defined as a first visit to a health professional (psychologist or psychiatrist) in the MMHS. The frequency of occurrence of consultations with the MMHS and of specific DSM IV diagnoses were presented as incidence rates (IR). IR were calculated as the number of new consultations divided by the person time at risk.

The person-time at risk for the non-exposed was defined as the time in days between the start of the observation period (September 1, 2008) or the date of hire (if this date occurred after September 1, 2008) and 1) a consultation with the MMHS, 2) the start of deployment, 3) the end of the observation period (November 15, 2010), or 4) the end of employment in the military, whichever one came first.

The person-time at risk for the exposed was defined as the time in days between the end of deployment to Afghanistan and 1) a consultation with the MMHS, 2) the start of a next military deployment, 3) the end of the observation period (November 15, 2010), or 4) the end of employment in the military, whichever one came first. In case of an additional deployment to Afghanistan, a new exposure period was started following the return from deployment. The number of deployments to Afghanistan was recorded and used as an exposure measure in analyses.

Military personnel that returned from deployment to Afghanistan before the start of observation were already exposed at the start of the observation period. The period between end of the deployment and start of the observation period in this study is known as the immortal person time (Rothman, Greenland, & Lash, 2008). These persons start contributing to the person time at risk for the exposed only from September 1, 2008. In other words, the period before September 1, 2008 was unobserved and was not included in the analysis (left truncation). For example, a person returning from Afghanistan on September 1, 2007, contributed to the person-time at risk for the exposed from September, 1 2008, or day 365, until maximally the day 805 after returning. A maximum follow-up of 805 days was chosen, as this was the maximum possible duration of follow-up for the non-exposed.

To compare crude incidences between exposed or non-exposed, or between other subgroups, incidence rate ratio (IRR) and incidence rate difference (IRD) were calculated. Cox proportional hazards models with left truncation were used to adjust for potential confounders of the observed differences in incidence and to explore temporal trends. Univariate analyses were followed by multivariable analysis, with forward selection of confounders. If inclusion of a potential confounder changed the main effect estimate by >10% the variable was retained in the model. The proportional hazards assumption was tested with the cox zph function in R (R Development Core Team, 2008). In case of non-proportionality, the time-trend was visualized by plotting the scaled Schoenfeld residuals against transformed time and the main exposure was included in the Cox proportional hazards model as a time-dependent covariate. To allow for non-linear function of the HR over time, quadratic and cubic functions of time and linear and restricted cubic splines were tested in the model. The choice for the best model was based on Akaike's Information Criterion (AIC). Robust standard errors are presented to allow for non-independence of multiple observation periods for individuals.

To assess the distribution of incidence among deployed personnel, additional analyses were performed, comparing various functional groups to non-deployed military personnel. Because personnel from units regularly operating off-base were more likely to be exposed to potentially traumatic events, e.g., violence, roadside bombings or suicide attacks, we additionally assessed whether military personnel from these units had a higher incidence of consultations with the MMHS. Units that were assumed to predominantly operate off-base were the Battlegroup (staff excluded), the Provincial Reconstruction Team and the military engineers. Finally, we assessed whether the number of previous deployments to Afghanistan was associated with a higher incidence of consultations with the MMHS.

Data analyses were performed with PASW Statistics (version 18) and statistical software R.

## Results

During the observation period, or part of this period, 57,878 military personnel were in active duty in the Netherlands. After applying the exclusion criteria, the study population consisted of 50,508 military (18,233 deployed, 32,275 non-deployed). The main reason for exclusion was participation in a (recurring) short deployment lasting <30 days (*N*=3,356).

The study population was responsible for 60,197 observation periods, equaling a total person time of 28,922,522 days; (27.7% exposed vs. 72.3% non-exposed). A single individual could account for more than one period, for example when a period without exposure was followed by a period of exposure. Of the 37,677 persons that were observed when not-exposed, 5,402 were eventually deployed to Afghanistan and therefore also included in the exposed group from the moment of their return.

The mean duration of a deployment to Afghanistan was approximately 4 months (118 days). The 22,520 observation periods for the exposed included 18,233 individuals. Demographic characteristics for the observation periods are presented in Table 1. During the study period 9,217 persons joined the Dutch military. The proportion of women and those <21 years was higher for the non-exposed compared to the exposed. Navy personnel and Military Police were underrepresented in the deployed cohort. Fewer deployed military ended their employment during the observation period than did non-deployed military.

**Table 1:** Characteristics of the 2008–2010 cohort of Netherlands Armed Forces at start of observation

	Observation periods not deployed to Afghanistan (“non-exposed”) *N*=37,677	Observation periods after deployment to Afghanistan (“exposed”) *N*=22,520	*P*
Sex; *N* (%)			Chi-square <0.0001
Male	33,728 (89.5)	21,147 (93.9)	
Female	3,949 (10.5)	1,373 (6.1)	
Service branch; *N* (%)			Chi-square <0.0001
Army	16,498 (43.8)	15,460 (68.7)	
Navy	8,533 (22.6)	1,478 (6.6)	
Air Force	6,773 (18.0)	4,793 (21.3)	
Marechaussee/military police	5,873 (15.6)	789 (3.5)	
Rank; *N* (%)			Chi-square <0.0001
Soldiers/corporals	17,974 (47.4)	9,486 (42.1)	
Non-commissioned officers	12,994 (34.5)	8,609 (38.2)	
Officers	6,709 (17.8)	4,425 (19.6)	
Age^a^ Median (IQR)^b^	25 (20–39)	28 (23–37)	Mann-Whitney <0.0001
Time in service^c^ Median (IQR)	3 (0–14)	6 (3–11)	Mann-Whitney <0.0001
End of employment during observation period^d^; *N* (%)	6,093 (16.2)	1,470 (6.5)	Chi-square <0.0001
Deployments to Afghanistan prior to the current deployment period; *N* (%)			
0	37,677 (100)	14,423 (64.0)	
1		5,823 (25.8)	
2		1,517 (6.7)	
≥ 3		757 (3.4)	
Deployments to other countries in history^e^; *N* (%)			Chi-square <0.0001
0	28,231 (74.9)	12,375 (54.9)	
≥ 1	9,446 (25.1)	10,145 (45.0)	

a Age in 2008

b IQR inter quartile range

c time in service at the start of the observation period

d loss to follow-up, censoring in cox-regression

e from 1978 until 2006.

A total of 1,906 first consultations with the MMHS took place during the observation period. The IR, IRR and IRD for consultations with the MMHS for military personnel deployed to Afghanistan compared to military personnel not deployed are presented in Table 2. The IRR for deployment was 1.60 (95% CI 1.45–1.75), indicating a 60% higher frequency of first consultations with the MMHS for military personnel deployed to Afghanistan during the study period compared to those not deployed.

**Table 2:** Military Mental Health Service (MMHS) utilization by Afghanistan-deployed personnel compared to non-deployed personnel, Netherlands Armed Forces 2008–2010

	Observation periods not deployed to Afghanistan (“non-exposed”) *N*=37,677	Observation periods after deployment to Afghanistan (“exposed”) *N*=22,520
Consultation with the MMHS	1,182	724
Person-time (days)	20,914,736	8,007,786
Incidence rate (IR) per 1,000 person months	1.71	2.73
Incidence rate ratio (IRR) (95% CI)		1.60 (1.46–1.76)
Hazard ratio (HR)^a^ (95% CI)		1.59 (1.45–1.75)
Incidence rate difference (IRD) (95% CI) per 1,000 person months		1.02 (0.80–1.25)

a Cox regression analysis.

**Fig. 1:**
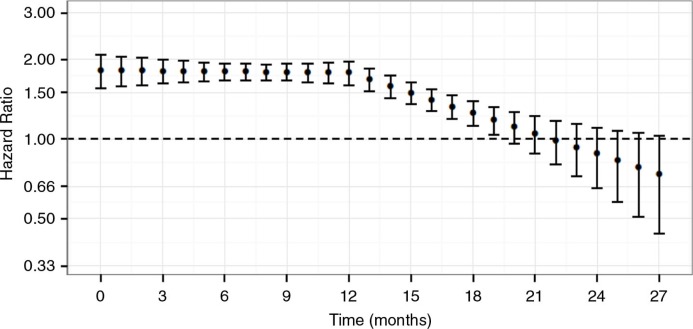
Time-varying adjusted hazard ratios (95% CI) for consultation with the Military Mental Health Service of Afghanistan-deployed personnel compared to non-deployed personnel, Netherlands Armed Forces 2008–2010.

The unadjusted HR was 1.59 (95% CI 1.45–1.75) for the exposed compared to the non-exposed. A multivariable Cox regression model containing the variables sex, military branch and time in service (in periods of 5 years) was used to correct for confounding. Because the assumption of proportional hazards was not satisfied (data not shown), an interaction term for time*exposure was added to the confounder-adjusted Cox regression model to allow time-varying hazards. Several models allowing the HR to change over time in a non-linear way were tested. The best fit model was a linear spline with a knot at 12 months. The change of the HR over time is shown in Fig. 1.

From Fig. 1, it can be concluded that the HR for a consultation with the MMHS is fairly constant around 1.80 in the first year and decreases in the second year following deployment. Likewise, the HR for a consultation with the MMHS for different subgroups of military personnel deployed to Afghanistan, or for specific DSM IV diagnoses varied over time. For practical reasons time-varying hazards were not explicitly modeled for each separate group and outcome, but the average HRs were estimated for the first and second years using a simple interaction-term; year-by-exposure.

The adjusted HRs for a consultation with the MMHS in the first and second year following deployment are presented for functional groups of military personnel deployed to Afghanistan (Table 3). The trend in time for these HRs was comparable to the trend of the total group. The HR is relatively high for military personnel deployed to a unit that predominantly operated off-base (“high-risk unit”). There were no significant differences in the incidence in military personnel following a first mission or after repeated missions (2nd, 3rd, 4th) to Afghanistan.

**Table 3:** Adjusted hazard ratio^a^ for consultation with the Military Mental Health Service in the first- and second-year post-deployment for different groups of Afghanistan-deployed personnel compared to non-deployed personnel, Netherlands Armed Forces 2008–2010

	Number of diagnoses for Afghanistan deployed military personnel (“exposed”)	Hazard ratio first year (95% CI)	Hazard ratio second year (95% CI)
Total group	724	1.84 (1.61–2.11)	1.28 (1.09–1.49)
Risk profile military unit^b^			
Low-risk units	170	1.29 (1.03–1.61)	1.04 (0.80–1.35)
High-risk units	554	2.13 (1.84–2.47)	1.40 (1.18–1.67)
Number of deployments			
After 1 deployment	470	1.84 (1.58–2.15)	1.33 (1.12–1.57)
After 2 deployments	178	1.78 (1.46–2.18)	1.07 (0.80–1.43)
After 3 deployments	53	2.05 (1.45–2.90)	1.33 (0.78–2.27)
After ≥4 deployments	23	1.92 (1.16–3.19)	1.85 (0.83–4.12)

a Cox-regression including time*exposure interaction term, adjusted for sex, military branch, and time in service

b high-risk units consist of military personnel that predominantly operated off-base (e.g., Battle Group excluding staff, Provincial Reconstruction Team, Military Engineers). All other units were defined as low-risk units.

The incidences of specific diagnoses made by the MMHS are compared in Table 4. The incidence of adjustment disorders and anxiety disorders, including PTSD, were significantly increased for military deployed to Afghanistan compared to the non-deployed personnel (Table 4). This was not the case for the diagnosis “mood disorders.” The difference in incidence for sleep disorders, impulse-control disorders or substance disorders were not included because their incidence was <10 per 10,000 individuals.

**Table 4:** Adjusted hazard ratio^a^ for consultation with the Military Mental Health Service in the first- and second-year post-deployment for specific psychiatric disorders (DSM IV) for Afghanistan-deployed personnel compared to non-deployed personnel, Netherlands Armed Forces 2008–2010

	Number of diagnoses for Afghanistan deployed military personnel (“exposed”)	Hazard ratio first year (95% CI)	Hazard ratio second year (95% CI)
Adjustment disorders	222	2.59 (2.02–3.32)	1.74 (1.30–2.32)
Anxiety disorders	108	2.22 (1.52–3.25)	2.28 (1.50–3.45)
Mood disorders	71	1.33 (0.90–1.97)	1.11 (0.68–1.82)
Posttraumatic stress disorder^b^	50	5.15 (2.55–10.40)	5.28 (2.42–11.50)

a Cox-regression including time*exposure interaction term, adjusted for sex, military branch, and time in service

b subgroup of the anxiety disorders.

## Discussion

The results of this study demonstrate that an 80% higher incidence of consultations with the MMHS was seen in Afghanistan-deployed Netherlands Armed Forces personnel in the first year following deployment when compared to personnel never deployed to Afghanistan (HR [95% CI] 1.84 [1.61– 2.11]). The incidence decreased during the second year following deployment HR 1.28 (1.09–1.49). In particular, the higher incidence was seen in military personnel deployed with a high-risk unit that predominantly operated off-base: HR 2.13 [1.84–2.47] and 1.40 [1.18–1.67] in the first- and second-year post-deployment, respectively. Previous deployment experience did not result in lower incidence of post-deployment consultations (Table 3). Adjustment disorders (HR 2.59 and 1.74), anxiety disorders (HR 2.22 and 2.28) and PTSD (HR 5.15 and 5.28) were more often diagnosed in deployed military personnel than in non-deployed military personnel. The frequency of diagnoses of mood disorders did not differ (HR 1.33 and 1.11).

Though a significantly higher incidence of consultations with the MMHS was observed, the absolute difference was small; on average only a single extra consultation with the MMHS per 1,000 military personnel per month was observed during the first 2 years in deployed military personnel following deployment to Afghanistan. Importantly, the incidence of consultations in deployed military personnel remained below the yearly incidence in the general population of approximately 0.2% (Schoemaker, 2011). Even though the impact of mental disorders for those involved is non-negligible and the relative increase in cases has implications for the workload of the MMHS, these figures do not support the view presented in the Dutch media that military involvement in Afghanistan has led to an epidemic of psychiatric illnesses in Dutch military personnel (Boon, 2011).

This study shows that adjustment disorders were the most common primary diagnoses in military personnel following deployment to Afghanistan; nearly one-third (222 from 724) of the diagnoses. Adjustment disorders are known to be a common problem following military deployment. More than 15 years ago it was concluded that approximately 20% of the Dutch military personnel suffered from adjustment problems after return from deployment (Bramsen, Dirkzwager, & van der Ploeg, 1997). The discrepancy between this study and the one mentioned above may suggest that, though a common occurrence, for the large part these adjustment problems are self-limiting and sufficiently mild that few military personnel visited the general practitioner and only those with severe symptoms were referred to the MMHS.

At first sight, the fivefold increase in PTSD diagnoses in the first 2 years following deployment to Afghanistan was a concerning high number. However, the total number of consultations with the MMHS resulting in a diagnosis of PTSD was not extremely high; 50 out of 754 (~7%) diagnoses. In approximately a quarter of the persons diagnosed with PTSD, symptoms started more than half a year after the potential traumatic event. According to Smid, Mooren, van der Mast, Gersons, and Kleber (2009), this delayed onset form of PTSD is seen more frequently among military personnel. Possibly, military group membership may initially create a feeling of safety and support that diminishes over time. Furthermore, fear for stigmatization of mental health problems in the military could lead to a delay in healthcare seeking behavior. The high HR of diagnoses of PTSD following deployment in this study, even during the second year, may in combination with the delayed onset form of PTSD, indicate higher numbers of PTSD diagnoses continuing for many years following deployment.

Relatively few military personnel received a diagnosis “substance disorders.” This could be explained by the fact that these diseases are not often recorded as the main diagnosis, though may be present in combination with other DSM IV diagnoses.

A large number of studies on the epidemiology of mental health disorders following military deployment are based on studies of US military personnel (Hoge et al., 2006; Milliken et al., 2007). Hoge et al. (2006) found that 35% of US military personnel accessed the Mental Health Services in the first year following deployment to Iraq. Based on screening using the Post-Deployment Health Assessment (PDHA) and the Post-Deployment Health Re-Assessment (PDHRA), self-report questionnaires and a brief interview with a primary health care professional, Milliken et al. (2007) concluded that more than 20% of active soldiers required mental health treatment, following deployment. Comparison of our results with these studies based on US military personnel should be made with caution. Due to the organization and financing/insurance policy of the US health care system, it may be beneficial for military personnel to visit a healthcare institution soon after return from deployment when the Veterans Administration will pay for it, which may lead to a higher chance of being diagnosed with a mental health disorder. Furthermore, the long duration of an average US military deployment (~1 year) together with recurrent deployments, with only a year between deployments, may account for the higher incidence in mental health problems for US military members (Thomas et al., 2010).

Research on British military personnel shows little difference in the presence of mental health problems in military personnel whether deployed to Iraq or Afghanistan or not (Iversen et al., 2009). Fear et al. (2010) demonstrated only a higher prevalence of alcohol misuse among deployed military members (OR 1.22) and military personnel from combat units reported a higher prevalence of PTSD (OR 1.87) compared to military personnel from support units. More recently, a study by Jones et al. (2013) confirmed that holding a combat role in Iraq or Afghanistan was associated with PTSD.

Different factors may have influenced our findings. The higher observed frequency of consultations with the MMHS for recently deployed personnel could partly be explained by the fact that colleagues and the officers monitor especially this group and thus mental health problems may be recognized early. Six months following deployment, all personnel receive a PDHRA questionnaire, a screening instrument used to assess both physical and psychosocial problems. If the filled-in questionnaire gives a cause for concern for developing serious psychological or physical health problems, a military physician could contact the military member and if necessary refer him to a health professional. Military physicians may be inclined to refer recently deployed military personnel to the MMHS more quickly, also because the military mental health care is highly accessible. On the other hand, the frequency of consultations with the MMHS for recently deployed personnel may be an underestimate of the true incidence, if there is a group of military personnel that avoid all possible care. These individuals may never consult a military health professional although they have serious psychological or physical health problems. It has been suggested that operationally active military personnel, in particular may avoid contact with the MMHS because they think this will negatively influence their military careers (Dohrenwend, Sloan, & Marx, 2008). Apart from fear of stigmatization, other so called “barriers to care” for military personnel of different Armed Forces are reported in the international literature (Gould et al., 2010; Hoge et al., 2004; Iversen et al., 2011; Sareen et al., 2007). For example, low confidence in the military health care system or interference of care visits with work hours and job workload. Unfortunately, quantitative data to assess to what extent the above mechanisms have led to over- or underestimation of the problem were unavailable for this study population.

There are some additional limitations that need to be considered in the interpretation of the findings. Although this study used routinely collected data, rather than data collected specifically for this study, we judge the quality of data to be good. All diagnoses were confirmed by psychiatrists or clinical psychologists trained in using the DSM-IV classification system. Clinical assessment is similar across all MMHS professionals and is recorded in the only information system available for them in the Dutch Armed Forces. Although the accessibility of the system was not optimal, this did not influence the accuracy of the data that was obtained. Because personnel registry data was used, the number of individual characteristics of military personnel was limited, precluding the analytic adjustment for potential confounding. However, it is unlikely that risk factors associated with the prevalence of mental health disorders (like genetic factors) were distributed differently among military personnel who were deployed versus those that were not, as all military are selected based on whether or not they are fit for deployment. Therefore, we feel that our results of relative differences are generally unlikely to be confounded. An exception may be confounded by civil status, because single military personnel may be more likely to be deployed more often. Having a partner typically offers some “protection” against mental health problems (Driessen, 2011).

Another limitation of this study is the availability of data only from 2008 onwards and not from the start of the ISAF deployment to Afghanistan in 2002. The follow-up time of somewhat more than 2 years was short, which prevents from drawing conclusions on the long-term effects of deployment. Also, this study only provides insight into mental problems seen by the MMHS. We do not have data about the consultation of mental health specialists working in a civilian setting, outside of the military system. Furthermore, it is currently unknown how often military personnel with mental health disorders only visit a military occupational social worker or army chaplain/padre but not a military psychiatrist or psychologist.

In conclusion, this study showed a higher risk for mental health problems resulting in a consultation with the MMHS following deployment to Afghanistan. To gain more insight in the grand total of mental health problems, visits to the *primary* military health care should also be taken into account. The registration of these consultations is provided through another computerized system, the system GIDS (Geneeskundig Informatiesysteem Defensie). This registry is not yet appropriate for epidemiological research (Leenstra, 2011). The findings reported in this paper underscore the necessity of an infrastructure that is prepared for the mental health needs of military personnel returning from deployment.
